# Does living in major towns favor institutional delivery in Somalia?

**DOI:** 10.3389/fgwh.2024.1216290

**Published:** 2024-07-25

**Authors:** Naima Said Sheikh, Ahmed M. Hussein, Shukri Said Mohamed, Abdi Gele

**Affiliations:** ^1^ Department of Public Health Science, Norwegian University of Life Sciences, Ås, Norway; ^2^ Department of Maternal and Reproductive Health, Somali Institute for Health Research, Mogadishu, Somalia; ^3^ Department of Health Services, Norwegian Institute of Public Health, Oslo, Norway

**Keywords:** Somalia, maternal health, institutional delivery, home birth, women's health

## Abstract

**Background:**

In developing countries, institutional delivery is a key proven intervention that reduces maternal mortality and can reduce maternal deaths by approximately 16%–33%. In Somalia, only 32% of births are delivered in a health facility with the assistance of a skilled healthcare provider. We aimed to investigate the factors hindering women from giving birth at healthcare facilities in major towns in Somalia, where most of the health facilities in the country are concentrated.

**Methods:**

A community-based health survey was carried out in 11 major towns in Somalia between October and December 2021. A structured and pretested questionnaire was used to collect data from 430 women who gave birth in the last five years. Women were recruited through convenient sampling. Descriptive statistics were used to summarize the data, and binary and multivariable logistic regression analysis was performed. Adjusted odds ratios (AOR) with 95% CI were estimated to assess the associations.

**Results:**

The overall prevalence of institutional delivery was 57%. Approximately 38% of women living in Mogadishu and 53% living in another ten towns give birth at home. Women who had poor knowledge of the importance of health facility delivery had nearly four times higher odds of delivering at home (AOR 3.64 CI: 1.49–8.93). Similarly, those who did not receive antenatal care (AOR 2.5, CI: 1.02–6.39) and those who did not receive a consultation on the place of delivery (AOR 2.15, CI: 1.17–3.94) were more likely to give birth at home. The reasons for home delivery included financial reasons, the long distance to the health facility, and the fact that it was easier to give birth at home.

**Conclusion:**

The study found that home delivery is high in major towns in Somalia and is associated with a lack of understanding of the importance of health facility delivery, not using ANC, and not receiving consultancy about where to give birth. Primary health care should strengthen information, education, and communication activities. Since the health care system in Somalia is overwhelmingly private, the government may consider access to free and within-reach ANC and health facility delivery for women and girls from families who cannot pay the ANC and childbirth delivery cost.

## Background

Over half a million women experience pregnancy-related deaths each year, with 99% of these deaths occurring in low- and middle-income countries (LMICs), where complications of pregnancy and childbirth are the leading causes of death among women of reproductive age (1). Approximately 85% of these deaths occur in Sub-Saharan Africa and South Asia, with countries experiencing scarce skilled health staff taking the most significant burden (2). The risk of maternal death has declined in some of these countries over the last decade, but there has been either no or diminutive improvement in some countries, including Somalia. More than 70% of all maternal deaths are due to preventable conditions such as hemorrhage, infection, unsafe abortion, hypertensive disorders of pregnancy, and obstructed labor (3, 4). To help improve maternal health, adequate access to healthcare during and after pregnancy is necessary (5). However, access to these services is not possible for some women due to underlying causes, such as poverty, inadequate, inaccessible, or unaffordable healthcare, unequal access to resources, the low status of women, and illiteracy (3).

Somalia is a Sub-Saharan African country characterized by more than three decades of civil war. The deterioration of Somalia’s public health system sector is due to the absence of government throughout the civil war and the subsequent years of widespread conflict (6). The maternal mortality rate (MMR) in Somalia is 692 per 100,000, which is one of the highest in the world (7). This is because the risk factors for MMR are abundant in Somalia. The armed conflicts in Somalia have forcibly displaced 0.9 million people (8), which is linked to a lack of- or limited access to maternal healthcare services due to safety, financial, and geographical restrictions (9). Furthermore, conflict makes using institutional delivery services very challenging, as conflicts hinder the movement of delivering mothers to functional health facilities (10). Conflicts also restrict access to health services, which can cause a dramatic increase in preventable deaths for mothers during pregnancy and childbirth (2). Recently published literature confirms that mothers who were exposed to armed conflict had a higher likelihood of experiencing maternal death (11). The contributing factors for the high MMR in Somalia include sociopolitical instability and ongoing wars, as well as other sociocultural challenges, poverty, long distances to the health facility, and health system limitations (5). There are also underlying factors, including a high rate of teenage pregnancy, as 20% of girls aged 15–19 have already had their first pregnancy, a high fertility rate (6.9 children per woman), a low use of modern methods of family planning (1%) and a high prevalence of female genital mutilation (99%) (7).

Reaching the aim of Sustainable Development Goals (SDGs) to reduce maternal and infant mortalities by 2030 (4) requires increased institutional delivery (5). Institutional delivery (ID) can reduce maternal deaths by 16% to 33% through the provision of safe delivery and can reduce complications related to and occurring during birth (12, 13). In Somalia, the Demographic Health Survey (SDHS) briefly mentioned the level of institutional delivery and showed that 32% of births in Somalia (including pastoralists and the rural population) are assisted by a skilled healthcare provider (7). Given the verified health benefits of institutional delivery, it is crucial to comprehend the range of factors that determine the choice of the place of childbirth. Prior studies on healthcare use and subsequent health facility delivery have highlighted a range of potential influences on a woman's tendency to seek care at a health facility, which includes younger maternal age, a woman's and husband's education, employment, contraception use, previous delivery at a health facility, utilization of antenatal care (14–16) and reduced health worker motivation, especially gynecologists and midwives in the case of Somalia (17). Despite this knowledge, the use of institutional healthcare delivery is failing in Sub-Saharan Africa due to a lack of access, distance, and insufficient equipment (5, 18).

Little is known about the factors related to the utilization of institutional delivery/skilled attendance at delivery in Somalia. To help fill this gap in knowledge, this study investigated the potential factors associated with institutional delivery among women living in major towns in Somalia, with the ultimate goal of providing relevant policy and programmatic recommendations as we prepare Somalia for achieving the SDGs’ health agenda for 2030.

## Methods

### Study design and population

A study survey was carried out in 11 major towns in Somalia between October and December 2021. The aim of this study was to investigate factors associated with institutional delivery in Somalia, which is a key intervention in averting the risk of maternal mortality due to childbirth-related complications. A total of 430 women of childbearing age were recruited through convenient sampling. The data were collected by eleven trained research assistants who were fluent speakers of the Somali language, and training was provided for data collectors on how to manage the data collection process. The overall activities of data collection were supervised and coordinated by the authors. The collected data were checked constantly for consistency, completeness, and relevance during the entire data collection process. We used a modified version of a questionnaire that was developed and used for a prior study among Somalis in Ethiopia (19). We largely amended the questionnaire to fit the research objectives and the urban population. Initially, we prepared the questionnaire in English, then translated it into Somali, and then back translated it into English to ensure the specificity of the questions. The Somali version was pretested before the actual data collection with eight women by trained research assistants. The necessary adjustments were made to develop the final version. A single population proportion formula was used to determine the sample size. The prevalence of institutional delivery in Somalia is unknown. Considering the prevalence of institutional delivery of 50%, a 95% confidence interval, a margin of error of 5%, and a 10% non-response rate, the final calculated sample size was 424, which we increased to 430.

### Study variables

Our independent variables were socio-demographics such as *age and sex* and included women who were between 15 and 50 years old, who gave birth in the last five years, and whether their *marital status* was married, unmarried, divorced, or widowed at the time of data collection. *Education level:* we asked women their education level as well as their husband's or child's father's education level, which was classified based on the highest attained educational level and grouped into; uneducated (meaning didn't attend school), “primary & middle school level” (≥9 years), “secondary school” (10–12 years), “university/university college” (13 ≥ 17 years) and “did not know” if women didn't know their partner's education level. *Employment status:* Employed (coded = 0) or unemployed (=1). Additionally, women who previously used health care services, including antenatal care, postnatal care, child vaccination, and/or who received consultation to where to give birth, were coded (0 = yes or 1 = no). Distance to the healthcare facility is determined by walking distance or whether they have to use transportation and, if so, whether they have access to their own transportation or whether they can use public transport to the clinic. We also asked women if they knew the benefits of giving birth at a healthcare facility rather than at home. Study area or “*Region*”: data were collected on women living in Mogadishu, Kismayo, Jowhar, Galkayo, Dhusomareb, Guriel, Beledwein, Bruao, Hargeisa, Afgoi and Bardere and shown in Supplementary Figure S1:

The dependent variable is the place of delivery and was categorized as “health facility” (coded: 0) or “home” (coded: 1).

### Operational definitions

*Institutional delivery* refers to childbirth that takes place in a healthcare facility, such as a hospital, maternity clinic, or health center, under the supervision of skilled health professionals like doctors, nurses, or midwives.

*Home delivery:* is when a mother gives birth at her home or another's home (neighbor, relatives, or family) or when a birth takes place outside the health institution.

*Skilled birth attendance:* The World Health Organization (WHO) defines skilled birth attendance as “an accredited health professional—such as a midwife, doctor or nurse—who has been educated and trained to proficiency in the skills needed to manage normal (i.e., uncomplicated) pregnancies, childbirth and the immediate postnatal period, and in the identification, management, and referral of women and neonates for complications. Traditional birth attendants, whether trained or not, are excluded from the category of “skilled attendant at delivery” (20).

*Traditional birth attendant (TBA):* a person with a specific task to aid the woman during labor and who learned their expertise by delivering newborns themselves or by training with other TBAs.

### Statistical analysis

The collected data were coded, entered, and cleaned in Excel software and were analyzed in Statistical Package for Social Science (SPSS) version 26.0. Descriptive statistics were performed to describe the data. As the level of education differed between women and their husbands, we grouped university and secondary education together for women while we coded separately for their husbands. We asked if the women knew the importance of the health facility delivery, with the response being coded as (yes; 0) or (no; 1). Descriptive analyses were conducted to describe the data. Binary logistic regression analysis was employed to examine the statistical associations between institutional delivery and each independent variable. Variables that showed significance at a *p*-value <0. 25 during bivariable analysis were entered into multivariable logistic regression to identify statistically significant variables. The Hosmer–Lemeshow test was used to check the model fitness for analysis. Crude odds ratios (ORs) and adjusted odds ratios (AOR) with 95% CI were estimated to assess the associations, and a *p*-value < 0.05 was considered statistically significant.

## Results

Table 1 summarizes the descriptive statistics and comparative analysis based on the place of childbirth. The study sample comprised 430 women of childbearing age. Of these, approximately 70% reside in Mogadishu, with the remaining 30% living in other towns. Among the total sample, 184 women (43%) delivered their last child at home. Specifically, about 38% of the women residing in Mogadishu and 53% of those living in other towns reported home births. The reasons for home delivery included that it was easier to deliver at home, financial reasons, and long distance to the health facility. Most women (82%) were younger than 35 years old, with approximately 69% being unemployed. Regarding education, 267 (62%) of the women and 182 (42%) of their husbands had no formal education. There was a significant difference in education between women who delivered at health facilities and those who delivered at home (*P* = 0.000). Approximately 77% of women delivered at home had no formal education. Similarly, 53% of women who delivered at home and 34% who delivered at a health facility had husbands with no formal education, which was significant (*P* = 0.001). Moreover, 96% of women who knew the benefit of a health facility delivery gave birth at a health facility (*P* = 0.000).

**Table 1:** Characteristics of the study sample and group differences by place of delivery among women in Somalia *(n = 430)*.

Variables		Health facility	Home delivery	Total	*P*-value
Age categories of women in years	15–25	94 (38)	69 (38)	163 (38)	0.482
	26–35	112 (46)	77 (42)	189 (44)	
	36 and over	40 (16)	38 (21)	78 (18)	
Civil status	Married	207 (84)	155 (84)	362 (84)	0.979
	Divorced or widowed	39 (16)	29 (16)	68 (16)	
Occupation	Employed	83 (34)	50 (27)	133 (31)	0.145
	Unemployed	163 (66)	134 (73)	297 (69)	
Women's education	High school and above	68 (28)	21 (11)	89 (21)	0.000
	Primary and middle school	53 (22)	21 (11)	74 (17)	
	Uneducated	125 (51)	142 (77)	267 (62)	
Husband's education	University level	66 (27)	15 (8)	81 (19)	0.000
	High school graduate	40 (16)	27 (15)	67 (16)	
	Primary and middle school	29 (12)	17 (9)	46 (11)	
	Uneducated	84 (34)	98 (53)	182 (42)	
	Do not know	27 (11)	27 (15)	54 (13)	
Distance to a health clinic	Walk distance	134 (54)	77 (42)	211 (49)	0.010
	Must use transport	112 (46)	107 (58)	219 (51)	
Number of children	1–3	108 (49)	79 (47)	187 (84)	0.762
	3–6	71 (32)	60 (36)	131 (34)	
	7 and above	40 (18)	28 (17)	68 (18)	
Sought antenatal care services	Yes	212 (86)	67 (36)	279 (65)	0.000
	No	34 (14)	117 (64)	151 (35)	
Received advice of the place of delivery	Yes	169 (78)	46 (56)	215 (72)	0.000
	No	47 (22)	36 (44)	83 (28)	
Know the benefits of health facility delivery	Yes	235 (96)	111 (60)	346 (81)	0.000
	No	11 (4)	73 (40)	84 (20)	
Wants to vaccinate their child	Yes	236 (96)	163 (89)	399 (93)	0.004
	No	10 (4)	21 (11)	31 (7)	
Wants to use postnatal care	Yes	236 (96)	158 (87)	394 (92)	0.001
	No	10 (4)	23 (13)	33 (8)	
Region of residence (state)	Benadir state (capital city of Somalia)	184 (75)	114 (62)	298 (69)	0.004
	Other states	62 (25)	70 (38)	132 (31)	

N, number, %, percentages.

*N* (%).

As shown in Table 2, after adjusting for age, marital status, region of residence, and employment status, women with low levels of education, as well as those whose husbands had low levels of education, had four times and five times higher, respectively, of giving birth at home. Women who needed transportation to access the nearest clinic had nearly twice the odds of home birth compared to those who could reach the facility on foot.

**Table 2:** Unadjusted and adjusted odds ratio (OR), 95% confidence intervals (CI) for factors associated with institutional delivery among women living in Somalia.

Dependent variable: place of delivery		Model 1 (unadjusted)	Model 2 (adjusted)
	Variables	OR [CI (95%)]	OR [CI (95%)]
Women's education	High school and above	1.00	** **
	Primary and middle school	1.28 [0.64–2.59]	1.33 [0.646–2.74]
	Uneducated	3.68 [2.13–6.35]	4.22 [2.371–7.52]
Husband's education	University level	1.00	** **
	High school	2.97 [1.41–6.25]	2.61 [1.22–5.58]
	Primary and middle school	2.58 [1.14–5.86]	2.71 [1.17–6.28]
	Uneducated	5.13 [2.73–9.66]	5.28 [2.76–10.09]
	Don't know	4.40 [2.03–9.54]	4.32 [1.94–9.58]
Distance to a health clinic	Walking distance	1.00	** **
	Must use transport	1.66 [1.13–2.45]	1.62 [1.09–2.41]
Number of children	1–3 children	1.00	** **
	3–6 children	1.16 [0.74–1.81]	
	7–13 children	0.96 [0.55–1.68]	
Sought antenatal care services	Yes	1.00	** **
	No	10.89 [6.80–17.43]	11.09 [6.79–18.10]
Received information about	Yes	1.00	
	No	2.81 [1.64–4.84]	2.99 [1.71–5.24]
Know the benefits of health facility delivery	Yes	1.00	
	No	14.05 [7.17–27.54]	14.02 [7.02–27.98]
Wants to vaccinate the child	Yes	1.00	
	No	3.04 [1.40–6.63]	2.80 [1.26–6.23]
Wants to use postnatal	Yes	1.00	
	No	3.44 [1.59–7.41]	3.26 [1.49–7.14]

Model 2: adjusted for age, marital status, region of residence, and employment. OR, odd ratio; CI, confidence interval.

Additionally, women who had poor knowledge about the benefits of institutional delivery were 14 times more likely to give birth at home (Model 1), while four times the odds of delivering at home after adjusting for women's and husband's education, age, region, marital status, and employment (model 3). Similarly, those who did not receive antenatal care (AOR 2.55, 95% CI: 1.02–6.40) and those who did not receive advice on the place of delivery (AOR 2.15, CI: 1.17–3.94) were more likely to deliver at home (Table 3).

**Table 3:** Unadjusted and adjusted odds ratio (OR), 95% confidence intervals (CI) for factors associated with institutional delivery among women living in Somalia.

Variables	Categories	Model 1: unadjusted OR [CI (95%)]	Model 3: adjusted OR [CI (95%)9
Distance to a health clinic	Walking distance	1.00	1.00
	Must use transport	1.66 [1.13–2.45]	1.18 [0.67–2.10]
Sought antenatal care services	Yes	1.00	1.00
	No	10.89 [6.80–17.43]	2.55 [1.02–6.40]
Received information about the place of delivery	Yes	1.00	1.00
	No	2.81 [1.64–4.84]	2.15 [1.17–3.94]
Know the benefits of a health facility delivery	Yes	1.00	1.00
	No	14.05 [7.17–27.54]	3.65 [1.49–8.93]
Wants to vaccinate their child	Yes	1.00	1.00
	No	3.04 [1.40–6.63]	2.39 [0.54–10.59]
Wants to use postnatal care	Yes	1.00	1.00
	No	3.44 [1.59–7.41]	1.03 [0.25–4.22]

Model 3: adjusted for women's and husband's education, age, town of residence, marital status, and employment. OR, odd ratio. CI, confidence interval.

## Discussion

This study investigated factors associated with institutional delivery among women living in 11 major towns in Somalia. The study showed that 43% of women in major towns in Somalia delivered at home. A recent study in Ethiopia found that the utilization of institutional delivery services in a fragile and conflict-affected setting in Ethiopia was 48.1%, which concords with our findings (10). In contrast, the SDHS showed that 78% of Somali women had delivered at home in past childbirths (7). The reason for the higher rates of home delivery in the SDHS study is that they did not stratify the variable “place of childbirth” into urban, rural, and nomadic, which obscured the difference between women who live in major towns, where healthcare facilities are concentrated, and rural and nomadic settlements where access to services are extremely limited. Access to healthcare varies considerably between urban and rural Somalia, as available health services are confined to major towns (21). Our findings are also in agreement with prior studies in Ethiopia, in which the odds of delivering at a health facility were seven times higher for women residing in urban vs. rural areas (22). In line with our findings, a recent study in conflict-affected areas of Ethiopia found that the use of institutional delivery services was associated with the maternal educational level of secondary school and above, antenatal care during the most recent pregnancy, being informed about birth preparedness and complication readiness and displacement of the respondents from their usual place of residence due to conflict (10). In Afghanistan, women with higher levels of education, from households in the upper two wealth quintiles, and who had any antenatal care were more likely to give birth in public or private facilities than at home (23). Given Somalia's very low institutional delivery rates, community outreach programs that provide information about delivery preparedness to pregnant women may elevate the country’s institutional delivery rate.

In our study, women who had no formal education (62%) had more than four times higher odds of giving birth at home than women with a secondary or higher level of education. This finding aligns with several studies that reported an association between women's education and health facility delivery (14, 15, 24). Our results are also in accordance with the SDHS, which shows that 68% of Somali women are illiterate, while only 9% of ever-married women are employed (7). A prior study revealed that the childbirths of women with no education are less likely to be assisted by skilled personnel (26%) than women with higher education levels (83%) (7). Our results also show that women living with an uneducated husband had five times higher odds of giving birth at home than women with a university/college education. In line with our findings, prior research reported an association between women's choice of childbirth place and their husband's level of education (25). A study in Nigeria revealed that women whose husbands had a tertiary education had an approximate 61% increased probability of a facility delivery (26). It is not only the education of women that is important in their choice of place of childbirth, but also that educated women had a greater probability of delaying marriage until the age of 18 years and of pregnancy until 20 years (27), as well as low fertility and high contraception use (28).

After controlling for the education level of women and their husbands, women with a poor understanding of the benefits of a health facility delivery had nearly four times higher odds of delivering at home than those with good knowledge. Similarly, those who did not receive consultancy on the place of childbirth were more likely to deliver at home. In line with our findings, a prior study in Zambia reported that women who know about the danger signs of pregnancy are more likely to deliver in a health facility than those without such knowledge (29). Similarly, women in rural Mali who were aware of possible pregnancy complications were more likely to give birth in a health facility (30). Knowledge of the importance of childbirth in a health facility determines women's birth preparedness and complication readiness, a strategy that has been globally endorsed as an essential component of safe motherhood programs to promote institutional delivery and the timely use of skilled maternal and neonatal care.

Our study showed that the need for transportation to reach the nearest health facility is associated with home delivery. Distance to health facilities is an obstacle to institutional delivery among women in Sub-Saharan African (14, 15). In our study, the distance to the health facility was measured by whether the health facility was within walking distance or if one must use transport to reach the nearest health facility. A study in Senegal associated a lack of transportation and residence more than five km from a health facility with giving birth at home (31). Moreover, in line with our findings, women in Eritrea who reside within 2 km of a health facility were 14 times more likely to deliver in a health facility than women who have to travel for more than two km to reach the nearest health facility (16). In Somalia, where most of the health facilities are private and, thus, more costly (21), pregnant women may not bear the multiple costs associated with seeking institutional delivery, including transport to the facility, and childbirth costs, including standard predelivery and postdelivery expenses such as facility fees and doctor fees, and other costs incurred due to their absence at home and children, which when combined may compel many women in major towns to give birth at home. On the other hand, the frequent armed conflict and rampant bandit roadblocks in Mogadishu, where most of the study participants reside, may discourage women from going to health facilities even within two km for childbirth, but instead, women may feel safe in giving birth at home. Furthermore, women may not consider taking transport to seek institutional delivery in areas such as Somalia, where childbirth is seen as a normal process, and there is a lack of social and cultural acceptability of facility services (32).

Finally, antenatal care (ANC), postnatal care utilization, and a willingness to vaccinate their child were all associated with health service delivery in our study. Our findings are in line with studies in Sub-Saharan Africa, thereby showing the importance of ANC utilization in health facility delivery. For instance, a literature review demonstrated the role of ANC in influencing health institution delivery, suggesting that all the elements of ANC are linked to higher health facility delivery (15). Similarly, a study in Uganda reported that women who used ANC were more likely to give birth in a healthcare facility than women who did not (33). Another study conducted in Ethiopia found that ANC follow-up was significantly associated with delivery in a healthcare facility, and as the number of antenatal care visits increased, the odds of a facility delivery increased (24). A study among Somalis in Ethiopia also shows that women who attended ANC had two times higher odds of delivering at a healthcare facility (19). It is widely accepted that ANC promotes the health of pregnant women by reducing the risk of adverse pregnancy outcomes while encouraging skilled birth attendance and postnatal care, as women who attend ANC are more likely to use these services than non-users (26, 34). A prior study found that birth preparedness messages provided during antenatal visits may increase the use of skilled attendance (increase the rate of institutional births) in areas where institutional births are low (35). The results from the systematic review showed that the utilization of community outreach services had the most significant effect on skilled birth attendance and antenatal consultation rates in conflict-affected countries (36).

This study has several limitations. This was a cross-sectional study; thus, there was no causal relationship. The study sample comprised women living in major towns where healthcare facilities are concentrated. However, the findings underscore that geographical proximity is insufficient for women to seek care from health facilities. We did not measure the distance to the health facility in kilometers but asked them if they had to use transport to access the facility. This may be a limitation because some people may choose to use transport even if the facility is within walking distance. Poverty, conflict-related factors, and other structural factors that were not considered in our study may also influence the result. In the sociocultural context, women encounter difficulties when accessing and using maternal health services. A qualitative study from Ghana found that women experienced difficulties conditioned by a religious obligation to maintain bodily sanctity through modest dressing and the avoidance of unlawful bodily exposure or contact with certain people, including male or alien caregivers, while some reflected issues related to lack of privacy, healthcare providers’ insensitivity and lack of knowledge about Muslim women's religious and cultural practices, and health information that lacked the cultural and religious specificity to meet Muslim women's maternity care needs (37). Our earlier study on the motivation of maternal health workers in Somalia showed that Somali male health workers had higher work motivation and higher education than female health workers, as also females were more representative in professions such as midwifery and gynecologists, which were the least motivated health workers in Somalia (17). Furthermore, we did not ask whether women who chose to deliver at home would receive help from a health worker at home or not. However, in Somalia, where there is an extreme shortage of trained midwives, doctors, and nurses, the chance of receiving professional assistance at home is limited. Even though that is the case, giving birth at home in Somalia with the presence of a trained midwife cannot compensate for the benefit of delivering in a healthcare facility. The reason for this is that if complications arise during childbirth, it is difficult in Somalia to find an emergency transport to the nearest hospital. Therefore, planned home delivery may not be a recommendable choice for women giving birth in Somalia.

## Conclusion

Despite these limitations, the research described in this paper has one main implication. The study found that home delivery was high in major towns in Somalia and was associated with a lack of understanding of the importance of a health facility delivery, not using ANC, and not receiving consultancy about where to give birth. This finding reflected that women in major towns in Somalia face challenges in some of the critical reproductive health services, such as antenatal care, delivery, health education, and counseling. In this light, information, education, and communication activities should be strengthened in primary health care to increase mothers’ awareness of risks associated with pregnancy and the importance of health facility delivery. Secondly, the reasons for home delivery included that “it was easier to deliver at home,”; “financial reasons,” and a “long distance to the health facility.” Since the health care system in Somalia is overwhelmingly private, the government should guarantee access to free and within-reach ANC and childbirth for women and girls from families who cannot pay the ANC and childbirth delivery costs. Further research is needed to understand the socio-cultural factors that hinder women from seeking institutional delivery. Multi-sectoral collaboration and innovative approaches such as public-private partnerships offer another avenue for creative solutions, leveraging the strengths of both sectors to improve maternal health outcomes. Collaborative efforts can involve mobile health clinics, telemedicine services, or voucher programs to increase urban women's access to ANC and delivery services. Community leaders’ support initiatives can be crucial in promoting maternal health by raising awareness, sourcing qualified staff for their networks, providing peer support, and facilitating access to healthcare services (6). Despite the fact that local and international NGOs bring valuable expertise, resources, and networks to the table, enabling targeted interventions and capacity-building efforts in maternal healthcare delivery, a recent study also indicated that the involvement of the diaspora community plays a crucial role in health service delivery in Somalia (6).

## Data Availability

The raw data supporting the conclusions of this article will be made available by the authors, without undue reservation.

## Supplementary material

Supplementary Figure S1 Study area. Created using Worldometer: https://www.worldometers.info/maps/somalia-political-map/.
