# Supplementary figures and images for: Does living in major towns favor institutional delivery in Somalia?
Source: Front Glob Womens Health. 2024 Jul 25;5:1216290. doi: 10.3389/fgwh.2024.1216290 (PMC11306125; doi:10.3389/fgwh.2024.1216290)

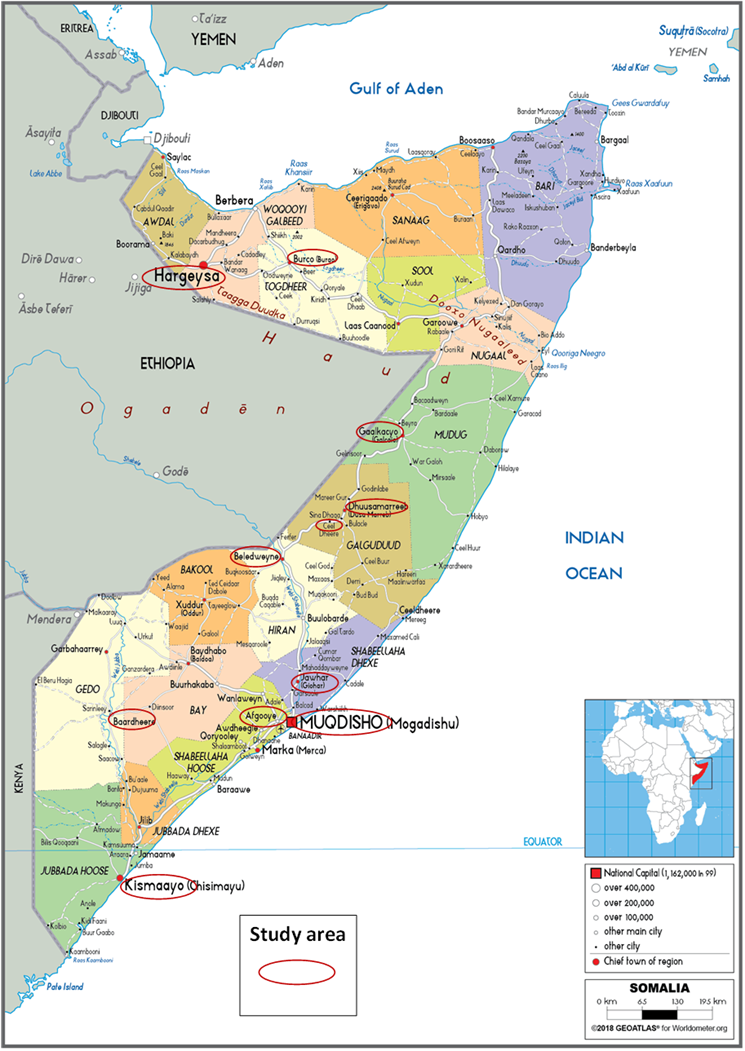

Supplement: Supplementary Figure S1 — Study area. Created using Worldometer: https://www.worldometers.info/maps/somalia-political-map/. [file Image1.png]
